# Object Detection Based on Lightweight YOLOX for Autonomous Driving

**DOI:** 10.3390/s23177596

**Published:** 2023-09-01

**Authors:** Qiyi He, Ao Xu, Zhiwei Ye, Wen Zhou, Ting Cai

**Affiliations:** School of Computer Science, Hubei University of Technology, Wuhan 430068, China; qiyi.he@hbut.edu.cn (Q.H.); aoxu_hbut@163.com (A.X.); zw_mmwh@hbut.edu.cn (W.Z.); caiting@hbut.edu.cn (T.C.)

**Keywords:** YOLOX, lightweight network design, object detection, attention mechanism, autonomous driving

## Abstract

Accurate and rapid response in complex driving scenarios is a challenging problem in autonomous driving. If a target is detected, the vehicle will not be able to react in time, resulting in fatal safety accidents. Therefore, the application of driver assistance systems requires a model that can accurately detect targets in complex scenes and respond quickly. In this paper, a lightweight feature extraction model, ShuffDet, is proposed to replace the CSPDark53 model used by YOLOX by improving the YOLOX algorithm. At the same time, an attention mechanism is introduced into the path aggregation feature pyramid network (PAFPN) to make the network focus more on important information in the network, thereby improving the accuracy of the model. This model, which combines two methods, is called ShuffYOLOX, and it can improve the accuracy of the model while keeping it lightweight. The performance of the ShuffYOLOX model on the KITTI dataset is tested in this paper, and the experimental results show that compared to the original network, the mean average precision (mAP) of the ShuffYOLOX model on the KITTI dataset reaches 92.20%. In addition, the number of parameters of the ShuffYOLOX model is reduced by 34.57%, the Gflops are reduced by 42.19%, and the FPS is increased by 65%. Therefore, the ShuffYOLOX model is very suitable for autonomous driving applications.

## 1. Introduction

Object detection is an important component of autonomous driving assistance systems, and the high accuracy and fast inference of object detection are very important for safe driving [1]. In recent years, as the number of vehicles has increased, the probability of traffic accidents and traffic congestion has gradually increased, and people’s traffic experience has become worse. Fortunately, the rapid development of graphics processing units and sensor technology has accelerated the development of vehicle detection [2]. Autonomous vehicles should be able to accurately identify targets such as vehicles, pedestrians, traffic lights, and road markings in front of them in unmanned driving situations and maintain real-time detection to ensure safety and correct decision making [3]. As an important part of the car, various sensors (such as lidar, ultrasonic radar, cameras, etc.) are used in autonomous vehicles [4]. Among these sensors, cameras can provide more visual information such as color, texture, shape, etc., and the cost is lower. Lidar can provide more accurate distance information, but the cost is higher and its detection capability is greatly reduced in rainy and foggy weather. Therefore, cameras are more cost effective [5]. For this reason, object detection algorithms based on visual cameras are widely used in visual tasks for autonomous driving. In general, a good object detection algorithm for autonomous driving should satisfy two conditions: high accuracy and real-time detection speed.

The original vehicle detection mainly relied on simple image processing by expert knowledge, using the symmetry of vehicles, shadows under vehicles, and other appearance features to construct corresponding vehicle detection methods. Hilario et al. proposed a method for detecting target symmetry using edge operators and applied it to practical detection tasks [6]. Ibarra-Arenado et al. used the knowledge of shadows under vehicles to obtain vehicle position information and used post-processing methods to detect vehicles [7]. However, these methods, which use vehicle appearance features for detection, are not robust in complex traffic scenarios and are affected by external factors such as weather, lighting, and road damage. Nowadays, vehicle detection methods can be divided into traditional vehicle detection and deep learning-based vehicle detection. Traditional vehicle detection mainly relies on manually extracted features combined with classifiers to distinguish between foreground and background. Dalal et al. proposed the classical HOG-SVM method to solve pedestrian and vehicle detection problems [8]. Wen et al. used the feature vector computed based on Haar-like features as the input parameter of the AdaBoost classifier for vehicle detection [9]. Karaimer et al. proposed a detection strategy that eliminates false judgments by subtracting the shape of moving targets and combining several consecutive frames [10]. However, the performance of these traditional vehicle detection methods depends on the quality of the manually extracted features, which requires researchers to have a certain reserve of expert knowledge when designing the features, and the design is time consuming. It should be noted that the generalization ability and accuracy of these algorithms are limited by the fact that the manually designed features are not powerful enough, resulting in poor detection performance in complex traffic scenarios. Deep learning-based vehicle detection can be divided into two-stage detectors and one-stage detectors. The detection accuracy of two-stage detectors is higher than that of one-stage detectors, but they are slightly inferior in terms of detection speed. For example, two-stage detectors such as R-CNN, Fast-RCNN, Faster-RCNN, etc., require a large number of proposal regions to be generated in advance, which greatly reduces the detection speed [11,12,13]. The drawback of low detection speed is obviously not suitable for fast response tasks such as autonomous driving. Of course, if there is a breakthrough in hardware performance, it can make up for the shortcomings of these detectors, so researchers still need to pay attention in the future. One-stage detectors such as YOLO and SSD refer to bounding box regression during classification, omitting the region proposal stage. These methods typically have fast computational speed and efficiency, but poor accuracy [14,15]. One-stage detectors, such as YOLOv2 and YOLOv3, strive to continuously improve the detection accuracy of the network [16,17]. In 2021, Ge et al. proposed YOlOX based on Anchor-free [18], which does not require predefined anchor points, but detects targets through dense feature extraction. The advantage of anchor-free networks is that they can reduce manual design processes while improving detection accuracy.One of the requirements for autonomous driving applications is to achieve a detection speed of more than 30 fps, while two-stage detectors cannot achieve real-time detection speed. Single-stage models can achieve real-time monitoring, but have slightly lower model detection accuracy. In 2017, Hu et al. proposed SENet [19], which introduced an attention mechanism to computer vision tasks for the first time. By obtaining the weights of each channel in the input feature layer, it improved the model’s ability to capture important information and reduce redundant network parameters. Subsequent methods, such as CBAM and ECANet, further promoted the application of attention mechanisms in computer vision and significantly improved model recognition accuracy [20,21].

Due to the memory limitations of on-board computers, there are also some deployment issues with vehicle detection models. The scale of high accuracy models is too large and not easy to deploy. ShuffleNet, proposed by Ma et al., is a lightweight network model that uses group convolution and channel transformation techniques to reduce the number of parameters and can be deployed on mobile devices, but the corresponding detection accuracy is also greatly reduced [22]. Therefore, relying solely on previous work has not achieved a good balance between accuracy, speed, and number of parameters. Specifically, in normal autonomous driving, misjudgments due to limited accuracy and untimely responses due to low response speed can both cause major road safety accidents.

In view of the problems existing in the above research, this paper proposes a lightweight one-stage detector ShuffYOLOX suitable for autonomous driving. Since the backbone network of YOLOX is too bloated and takes too long during inference, we propose a lightweight backbone network ShuffDet to improve the inference speed of the model and reduce its complexity. To compensate for the loss of accuracy, we introduced the ECA attention method in the enhanced feature extraction part to improve the model’s ability to capture features. Therefore, our proposed ShuffYOLOX has faster recognition speed and lower model complexity while maintaining accuracy. We validated our proposed method on the KITTI dataset and compared it with the base network (YOlOX). The detection speed of our model increased by 65%, the number of parameters decreased by 34.57%, and the accuracy remained basically the same. In autonomous driving, the vehicle detection task should comprehensively consider the key points of model accuracy, speed, and number of parameters to ensure the safety of intelligent connected vehicles during driving. Our proposed algorithm strikes a balance between accuracy and detection speed, and is suitable for autonomous vehicle detection. The main contributions of this paper include:

1. We propose a lightweight one-stage object detection model called ShuffYOLOX based on YOLOX for autonomous driving. To extract target features in autonomous driving scenarios, we use an improved lightweight model, ShuffDet, to replace the original CSPDark53 network in YOLOX. This greatly reduces the complexity and parameters of the model and significantly improves the inference speed of the model.
2. By adding ECA modules to the path aggregation feature pyramid network (PAFPN) structure, we have improved the network’s ability to capture information between channels.
3. We conducted experiments on the KITTI dataset to evaluate ShuffYOLOX and compared it to YOLOX as a baseline. The results show that our proposed model achieves similar accuracy to the baseline network, but with a 65% increase in inference speed and a significant reduction in complexity.

The organization of this paper is as follows: Section 2 provides a detailed introduction to the YOLOX network, network lightweighting criteria, and the ECA attention mechanism. Then, Section 3 elaborates on the improvement details of the ShuffYOLOX algorithm. Section 4 provides a detailed description and analysis of the experimental steps. Finally, in Section 5, the work is summarized.

## 2. Related Work

### 2.1. YOLOX

YOLOX [18] is a convolutional neural network algorithm used for object detection. Its network structure consists of three parts: a backbone network, an enhanced feature extraction network, and a detection network, which are used for feature extraction, enhanced feature extraction, and multi-scale prediction [23], respectively. The backbone network adopts CSPDarkNet53 [24] as the basic feature extraction network, which is an improved version of DarkNet53 that introduces the Cross Stage Partial (CSP) structure to improve the efficiency and accuracy of the model. The main idea of CSPDarkNet53 is to divide each residual block into two parts: the main branch and the side branch. The main branch is responsible for convolutional operations and feature extraction, while the side branch reduces the number of channels through 1x1 convolution. In this paper, this structure is represented by the Dark block. This design can reduce the number of parameters and computational complexity in the model while maintaining its accuracy.

Traditional YOLO networks, such as YOLOv1, YOLOv2, and YOLOv3 [14,16,17], grid the feature layers and generate multiple prior boxes for each feature point. The YOLO detection head detects whether each prior box contains an object and its category, and fine-tunes each prior box for detection results for each feature point. In contrast, YOLOX, as an upgraded version of the YOLO series, uses anchor-free technology for object detection. The anchor-free technology predicts object bounding boxes using the detection head, instead of fixing the size and shape of the bounding boxes using anchor boxes like traditional YOLO. This technology can detect objects of different sizes more accurately and converge faster. YOLOX incorporates the advantages of anchor-free technology into the network. YOLOX is currently one of the best object detection networks, and its excellent detection performance and recognition accuracy benefit from several innovative indicators, such as the simOTA dynamic matching mechanism that solves the problem of imbalance between positive and negative samples, and the anchor-free technology that can accelerate convergence. In the design of the detection head for classification and regression, YOLOX is different from the traditional YOLO versions. It adopts a decoupled approach and divides it into two parts: one part is classified through two 3 × 3 convolution blocks, and the other part is regressed through two 3 × 3 convolution blocks.

**Figure 1:**
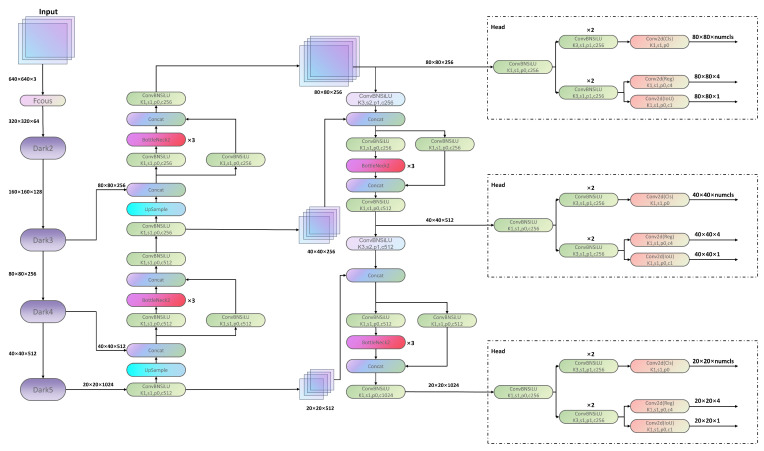
The overall network structure of YOLOX.

The YOLOX network amalgamates the merits of the YOLO series by employing the Focus and CSPNet architectures in the backbone network to efficiently extract and analyze image features, while integrating a decoupled structure within the detection head. The overarching architecture is depicted in Figure 1. Diverging from traditional anchor-based detection algorithms, YOLOX adopts an anchor-free design and incorporates the simOTA dynamic matching mechanism, leading to marked improvements in detection accuracy, faster convergence rates, and reduced network parameter redundancy. In the realm of single-stage object detection, the YOLOX algorithm exhibits commendable performance, achieving a notable 57.8 FPS operating speed on Tesla V100 hardware. However, YOLOX still harbors a number of modules with considerable parameter redundancy, exemplified by the Spatial Pyramid Pooling (SPP) module in the Dark5 module, aimed at mitigating challenges associated with varying input feature map sizes while bolstering the network’s receptive field and feature extraction capacities. Nevertheless, the 9 × 9 and 13 × 13 pooling layers within the SPP module entail substantial parameter calculations, culminating in parameter redundancy and elevated MAC. Given resource constraints, achieving real-time performance on autonomous driving devices remains an ongoing challenge.

### 2.2. Lightweight Criterion of Network Structure

Various approaches exist for achieving network lightweighting, including techniques such as knowledge distillation and pruning to compress models, as well as designing lightweight architectures within the network structure. For example, SqueezeNet [25] replaces large convolutional kernels with small ones to reduce the number of parameters, while MobileNetV1 [26] uses depth-wise separable convolution to further reduce parameter counts during the convolution process. ShuffleNetV1 [22] employs grouped convolution and channel shuffling to considerably expedite the forward inference process of the model. Especially in the ShuffleNetV2 [27] paper, Ma et al. propose that using Flops as the exclusive metric for computational complexity is not accurate, as it could result in numerous suboptimal designs. For example, MobileNetV2 [28] is much faster than NASNet-A [29] but has similar Flops. Flops do not take into account the memory access cost (MAC). In the context of grouped convolution, the MAC can dominate a significant portion of computation time. Hence, Ma et al. establish the initial lightweight criterion through extensive experiments: minimizing the MAC by having the input channel number equal the output channel number during convolution.

**Figure 2:**
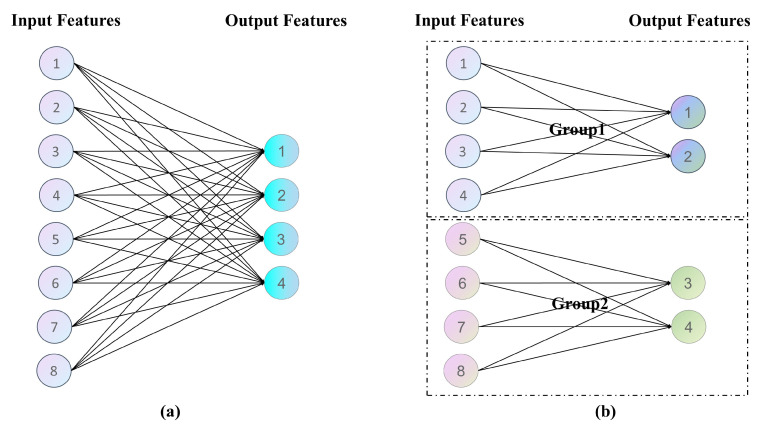
Group convolution structure diagram, (**a**) is the standard convolution, (**b**) is the group convolution.

Group convolution is currently one of the core methods for reducing the number of parameters in the convolution process. Its principle is to divide the input feature map into several groups, and each group’s channels perform convolution separately, and finally concatenate the results of each group’s convolution. The main advantage of group convolution is that it can reduce computational complexity and memory usage while reducing the number of parameters while maintaining the convolution effect. The structure of group convolution is shown in Figure 2. However, using group convolution will lead to an increase in memory access cost. This is because in a scenario with a fixed number of Flops, more channels are required to enhance the network’s information capacity, resulting in an increase in the MAC. Thus, the second lightweight criterion emerges! Extensive grouping will lead to a rise in the MAC.

Network segmentation involves breaking down a large convolution kernel into smaller ones and applying them to different regions of the input feature map. For instance, in the GoogLeNet [30] series, each network block extensively employs a multi-path structure. While network segmentation can reduce computational complexity and the number of parameters, it also has implications for parallel computation. Specifically, network segmentation introduces dependencies between the input and output of convolutional operations, necessitating a prescribed execution sequence for these operations. Such dependencies decrease parallelism, as certain convolutional operations must await the completion of preceding operations before initiation. Furthermore, applying network segmentation to larger convolutional kernels such as 7 × 7 or 9 × 9 kernels might exacerbate parallelism issues. These kernels themselves entail significant computation, and their division into smaller kernels for computation introduces more computational dependencies and decreases parallelism. Although this fragmented structure has demonstrated the potential to enhance model accuracy, it may reduce efficiency due to its adverse impact on GPU parallel computation. Thus, the third lightweighting criterion for network structure dictates that segmentation operations impede parallelism, thereby warranting prudence in their usage, particularly on larger convolutional kernels.

In the process of constructing neural networks, element-wise operations are unavoidable, which perform the same operation for each element of a tensor. For example, the ReLU activation function and Batch Normalization [31] layer in convolutional neural networks are both element-wise operations. Although the Flop of element-wise operations is small, they have a relatively heavy MAC, because the main element operation usually requires storing a large number of intermediate results in memory. When performing a ReLU operation on a layer in the network, the input and output feature maps need to be stored. If the size of the tensor is large, the memory overhead of these intermediate results may be very large, even exceeding the limit of GPU memory. In addition, element-wise operations usually require the same operation for each element, which may result in inefficient calculations. Element-wise operations are often a serial operation, meaning that each element needs to wait for the previous element to complete before performing the calculation. This means that in parallel computing, element-wise operations may become a bottleneck, leading to a decrease in parallel efficiency. Ma et al. conducted experiments on the bottleneck unit of ResNet [32] (1 × 1 convolution layer followed by a 3 × 3 convolution layer and a 1 × 1 convolution layer, with ReLU and shortcut connections), and found that deleting the ReLU and shortcut operations resulted in about 20% acceleration on both GPU and ARM. Therefore, the fourth criterion for lightweight network structure is not to ignore the impact of element-wise operations on memory and computation time.

### 2.3. ECA Attention Mechanism

The attention mechanism plays a pivotal role in the field of deep learning and has achieved remarkable breakthroughs in computer vision and natural language processing. In neural networks, the attention mechanism refers to concentrating limited computational resources on more critical tasks to solve the issue of information overload, thereby enhancing task processing efficiency and accuracy. While more model parameters usually indicate greater expressive capacity and larger information storage, they can also lead to information overload. Consequently, the introduction of attention mechanisms enables the focus on crucial information for the present task amidst a multitude of input data, reducing attention towards other details and even eliminating irrelevant data. This is akin to the human visual attention mechanism, wherein the global image is scanned to identify the target area of interest, followed by allocating heightened attention resources to that region, thereby obtaining more intricate information pertinent to the target while disregarding unrelated data. This mechanism empowers us to swiftly select high-value data using limited attention resources.

In essence, the fundamental function of the attention mechanism is to allow neural network layers to concentrate more on local features containing vital information, thereby enhancing the performance of neural network layers in feature extraction. When utilizing convolutional layers to extract image information, if network layers can adaptively prioritize significant local details, the feature extraction process of neural networks becomes notably more efficient.

In the field of computer vision, the application of attention mechanisms can be classified into two types: spatial attention mechanisms and channel attention mechanisms. SENet, proposed by Hu et al. in 2017, is a typical implementation of the channel attention mechanism, and it was also the champion model of the last ImageNet [33] competition. The basic mechanism of SENet [19] is to improve model performance by analyzing the correlation between different feature channels. In contrast to attention mechanisms that only focus on the correlation between feature channels, CBAM [20] proposed by Woo et al. integrates channel attention and spatial attention mechanisms to focus on deeper semantic information. Although CBAM can improve the performance of deep convolutional networks, the complex attention mechanism module also increases model complexity. To strike a balance between performance and complexity, Wang et al. conducted in-depth research on channel attention mechanisms and proposed ECANet [21] based on a non-dimensional local channel interaction strategy. ECANet initially acquires a feature map of size 1 × 1 × C through global average pooling, with C representing the number of feature channels. Subsequently, a one-dimensional convolution is performed using k convolutional kernels to extract feature information, where k is adaptively determined based on the channel count as illustrated in Equation (1). Ultimately, a sigmoid activation function is employed to generate the final channel weights. As the convolutional layer employs one-dimensional convolution, this module encompasses only a limited number of parameters while delivering notable performance enhancement.

(1) $$k=Y\left(C\right)={\left|\frac{\log_{2}\left(C\right)}{\gamma }+\frac{b}{\gamma }\right|}_{\mathrm{odd}\;}$$

In this equation, C represents the number of feature channels, ${|*|}_{\mathrm{odd}\;}$ denotes the nearest odd integer, and γ = 2, *b* = 1.

**Figure 3:**
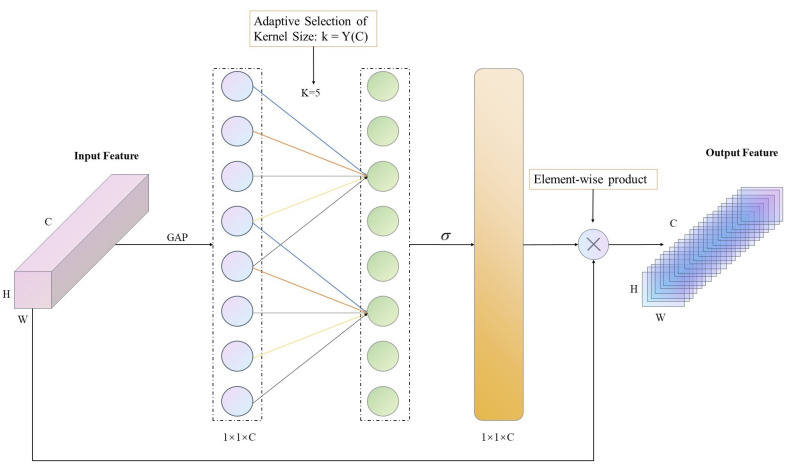
The structure diagram of the ECA module.

Due to the use of a path aggregation feature pyramid network (PAFPN) [34] in ShuffYOLOX, important feature information may be lost during the feature fusion process. To address this issue, attention mechanisms can be employed to improve the feature fusion module and obtain more effective detection feature maps. By enhancing the weight of important features and suppressing irrelevant ones, the problem of feature information loss during feature fusion can be mitigated. Considering the impact of attention mechanisms on model complexity, this paper adopts the ECA attention mechanism module (as shown in Figure 3) to improve detection performance while reducing model complexity. The ECA attention mechanism module can effectively solve the problem of information loss during feature fusion while having a relatively small impact on model complexity.

## 3. ShuffYOLOX: A Faster and Lighter Architecture

**Figure 4:**
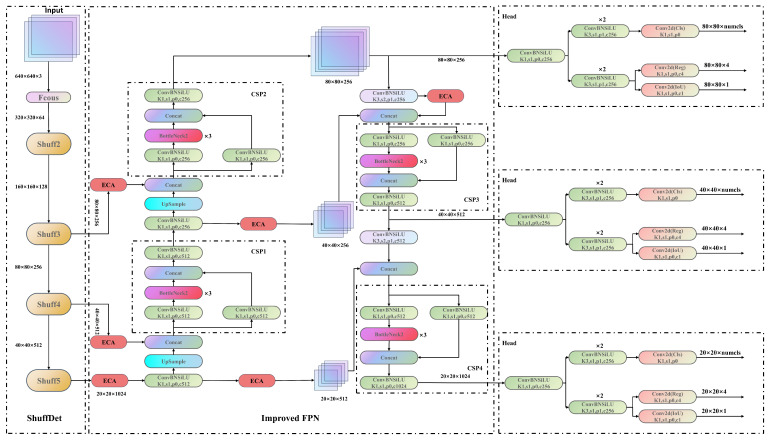
The overall network structure of ShuffYOLOX. The backbone is replaced with ShuffDet, PAFPN is a feature pyramid network. ECA attention mechanism was added in improved PAFPN.

ShuffYOLOX is a lightweight object detection network model based on the YOLOX framework. The backbone network of the YOLOX model, CSPDarkNet53 [24], is too large to be deployed on autonomous driving terminal devices. To address this issue, we proposed a lightweight backbone network, ShuffDet, to replace CSPDarkNet53. In designing ShuffDet, we followed four network structure lightweighting criteria. Moreover, to address the problem of feature information loss during feature fusion in the path aggregation feature pyramid network (PAFPN) of YOLOX, we introduced six lightweight attention mechanism ECA modules into the PAFPN to further improve the efficiency of feature extraction in the model. ShuffYOLOX can meet the deployment requirements of autonomous driving terminal devices and can effectively detect and recognize traffic road objects such as pedestrians, vehicles, and traffic signs. The structure of ShuffYOLOX is shown in Figure 4.

### 3.1. ShuffDet

**Figure 5:**
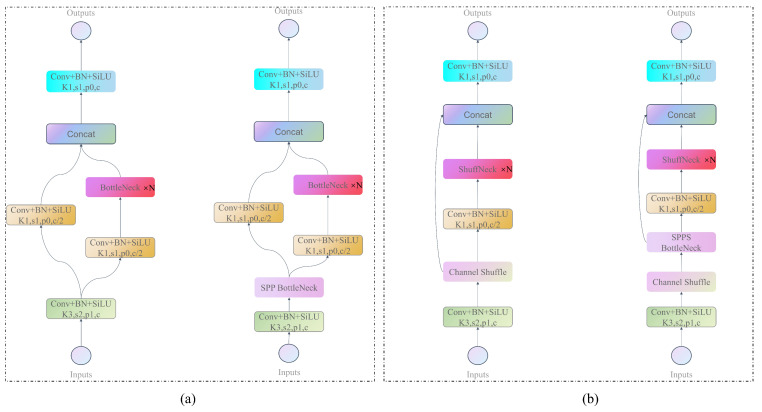
Dark and Shuff blocks structure diagram. (**a**) is the Dark, (**b**) is the Shuff.

The CSPDarkNet53 backbone network of YOLOX primarily integrates compound residual structures of CSPLayers, where each block comprises convolutional layers and residual blocks, termed Dark blocks in preceding sections. The computation within Dark modules involves extensive element-wise operations and residual structures, significantly inflating the MAC (As depicted in Figure 5a). Hence, to tackle the trade-off between accuracy and speed, we have innovatively introduced the ShuffDet structure (As depicted in Figure 5b), which is designed based on lightweight design principles. It considerably reduces model complexity while simultaneously maintaining accuracy. Following a straightforward 1 × 1 convolutional operation, the Shuff module undertakes channel shuffling, segmenting the feature map into two segments along the feature channels. The primary segment encompasses convolutional layers and Shuff bottlenecks, while the remaining portion remains untouched, directly linking with the main segment along the feature channels. In comparison to Dark blocks, Shuff blocks adhere entirely to lightweight standards. Additionally, within each Shuff block, half of the feature channels traverse the module and directly merge with the subsequent module, a concept akin to feature reutilization. With the connection between adjacent layers being stronger compared to connections between other layers, dense inter-layer connections could yield feature redundancy. Consequently, within the Shuff module, we directly concatenate half of the feature channels with the main segment without any modifications [35].

**Figure 6:**
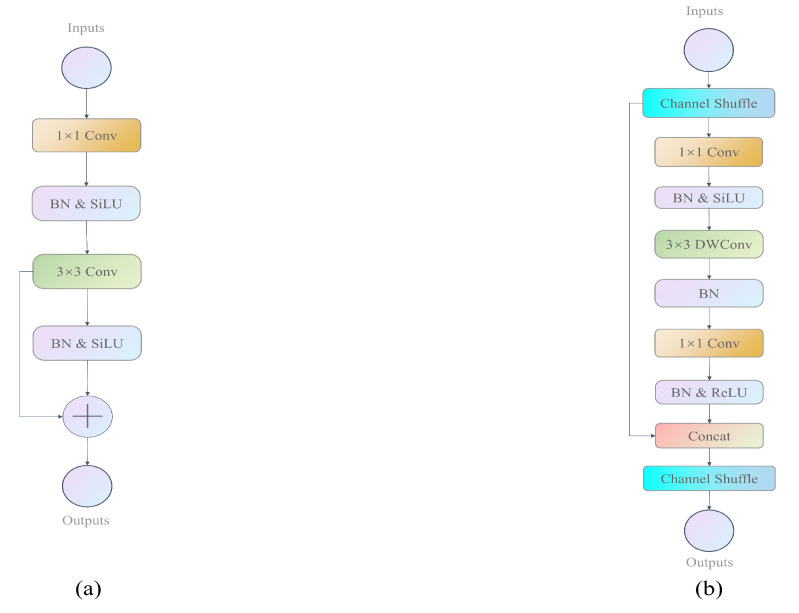
Residual blocks structure diagram, (**a**) is the bottleneck of YOLOX, (**b**) is the Shuff bottleneck.

The CSPDarkNet53 backbone network extensively employs residual bottleneck [36] structures, as shown in Figure 6a. However, these structures significantly increase the MAC for lightweight networks, which cannot be ignored. Therefore, in order to achieve higher model capacity and efficiency, we improved these bottleneck structures by using channel shuffle operations to divide the input feature maps into two parts. The backbone part is processed by two 1 × 1 convolutions and a 3 × 3 depthwise separable convolution layer in the middle, while the other part is left untouched, and the two parts are stacked together, as shown in Figure 6b. We named this improved bottleneck structure the “Shuff bottleneck”. The Shuff bottleneck is designed according to lightweight design principles, where the input channel number is the same as the output channel number, conforming to principle one. The element-wise “add” operation in the shortcut connection is replaced by a stacking operation, and there is no ReLU activation after the depthwise separable convolution, conforming to principle four. To avoid increasing the MAC cost, grouped convolution is not used in the Shuff bottleneck block, conforming to principle two.

**Figure 7:**
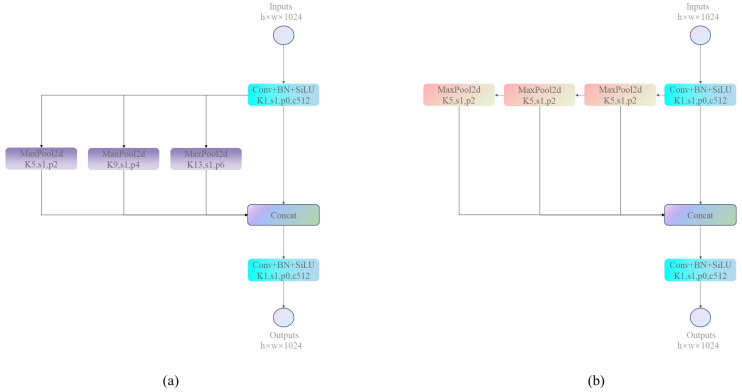
SPP blocks structure diagram, (**a**) is the SPP block of YOLOX, (**b**) is the SPPS block of ShuffYOLOX.

Within the Dark5 module, the Spatial Pyramid Pooling (SPP) module is employed to counteract the issue of information loss and distortion arising from disparate input feature map sizes. Concurrently, it amplifies the network’s receptive field and feature extraction competence. The SPP module engages multiscale pooling operations to convert input feature maps of varying sizes into standardized feature vectors. In the context of Dark5, the SPP module adopts three distinct pooling ratios: 5 × 5, 9 × 9, and 13 × 13, as shown in Figure 7a. Nevertheless, the 9 × 9 and 13 × 13 pooling layers within the SPP module entail substantial parameter calculations, resulting in parameter redundancy and an augmented MAC. Thus, we introduce a sequential SPPS structure. Combining three 5 × 5 pooling layers attains equivalent outcomes to three parallel pooling layers of distinct scales, as shown in Figure 7b, while drastically reducing computational load. Leveraging smaller pooling layers and a sequential structure, the sequential SPPS module achieves greater efficiency while retaining parity with the conventional SPP module.

### 3.2. Imporved Feature Pyramid Network

In object detection tasks, feature maps of different scales contain object information of different scales. To fully utilize this information, feature fusion is needed between feature maps at different levels. YOLOX introduces a path aggregation feature pyramid network (PAFPN) as the feature enhancement module, which enhances the output feature maps of Dark3, Dark4, and Dark5 in the backbone network. In order to fuse the three feature maps of different scales, their sizes need to be unified. Therefore, the PAFPN first uses a top-down upsampling method to enlarge the size of the deep feature map to match that of the shallow feature map, using bilinear interpolation, so that they can be fused. Then, through bottom-up convolution or pooling operations, the feature map is gradually downsampled from shallow to deep layers to extract more semantic information. During the top-down and bottom-up process, the PAFPN fuses feature maps at different levels through cross-layer connections. Specifically, during the top-down process, the PAFPN fuses the deeper feature map with its next lower-level feature map to obtain a new feature map. During the bottom-up process, the PAFPN fuses the lower-level feature map with its next higher-level feature map to obtain a new feature map. This combination of top-down and bottom-up operations helps the network extract better features.

**Figure 8:**
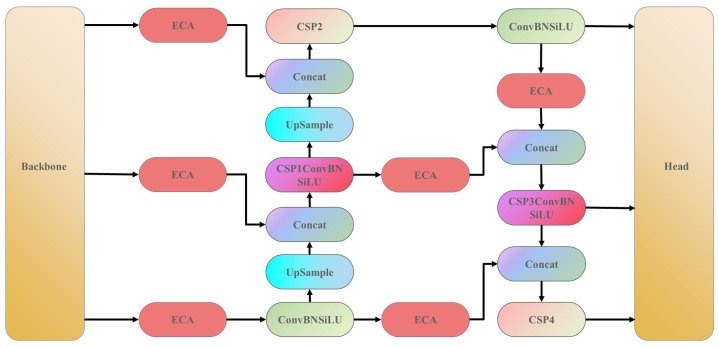
Improved PAFPN structure diagram. ECA is a lightweight attention module.

The Shuff blocks in our backbone network contain a large number of 1 × 1 convolution operations, resulting in feature maps with rich semantic information. However, in the feature pyramid structure, upsampling and downsampling operations are unavoidable. These operations can change the size and resolution of the feature maps, potentially leading to the loss of original semantic information. In particular, downsampling operations often lose local detailed information through pooling or convolution operations, making the loss of semantic information in certain regions of the feature maps more significant. To address this issue, we introduced the ECA attention module in the PAFPN. This module aims to enhance the feature extraction network to effectively extract key features from the feature maps and improve model accuracy. We introduced six ECA attention blocks in the PAFPN, located before the feature concatenation operation, as shown in Figure 8. Although we added multiple ECA attention blocks, they only perform Conv1D operations, adding very few parameters to the network. Experimental results show that the ECA module can significantly improve the model’s ability to capture key features with very few added parameters.

## 4. Experiments and Results

### 4.1. Dataset and Experimental Environment

The KITTI [1] dataset is a dataset specifically designed for object detection and tracking tasks in autonomous driving scenarios, created in collaboration between the Karlsruhe Institute of Technology (KIT) in Germany and the Toyota Technical Center in the USA. The dataset contains multimodal data from multiple sensors, such as lidar, cameras, GPS, and annotation information related to them, including the categories, locations, sizes, and orientations of the objects in the images. The KITTI dataset includes various tasks such as object detection, semantic segmentation, 3D object detection, and 3D object tracking, among which object detection is one of the most commonly used tasks. The goal of this task is to detect various objects such as traffic signs, vehicles, and pedestrians in the images and provide information such as their positions and sizes. The KITTI dataset contains real-world image data collected in various scenes such as urban, rural, and highway, with varying degrees of occlusion and truncation, which is beneficial for verifying the effectiveness of ShuffYOLOX in this paper. The dataset consists of 7481 training samples and 7518 testing samples, including 8 different types of objects, namely, cars, trucks, vans, pedestrians, sitting pedestrians, bicycles, trams, and other objects. For ease of study, we reclassified the dataset into five categories: car, tram, truck, van, and pedestrian. In addition, the 7481 training samples were split into training and validation sets with an 8:2 ratio for the experiments in this paper.

In this paper, the Pytorch deep learning framework is used to design and implement the algorithm on the Ubuntu 22.04.2 system. We trained the model using the device parameters shown in Table 1.

During the training process, the optimization of training parameters was performed using stochastic gradient descent [37]. The batch size was set to 4, and the number of workers was set to 5 to load data using multiple threads. The momentum was set to 0.92, and the weight decay coefficient was set to 0.0005. In addition, we used a dynamic learning rate during training, initializing the learning rate to 0.01, and gradually decreasing it through the cosine learning rate strategy [38]. All models were trained for 300 epochs. Each epoch corresponds to training the entire training dataset once through the network. The corresponding loss values were recorded for each epoch.

### 4.2. Evaluation Index

To evaluate the effectiveness of the proposed model, we used the evaluation standards of mainstream object detection models to evaluate our model. Frames per second (FPS) were used as the speed evaluation metric for the model, while mean average precision (mAP) was used as the accuracy evaluation metric for the model.

Precision and recall are metrics for evaluating the performance of classification models. Precision measures the proportion of true positive samples in the predicted positive samples of the model, and can be represented by Formula (2). Recall measures the proportion of true positive samples that are predicted correctly by the model, and can be represented by Formula (3). F1 score is the harmonic mean of precision and recall, and can be represented by Formula (4). It is used to comprehensively evaluate the results of precision and recall, with a value range of 0 to 1, where a value closer to 1 indicates better model performance.

(2) $$P=\frac{TP}{\left(TP+FP\right)}$$

(3) $$R=\frac{TP}{\left(TP+FN\right)}$$

(4) $$F1=\frac{2PR}{\left(P+R\right)}$$

Average precision (AP) takes into account the precision and recall of the model on each class and can be represented by Formula (5). It is an intuitive evaluation criterion for evaluating the accuracy of object detection models. The mean average precision (mAP) represents the average of AP for all classes in the evaluation dataset, which can be represented by Formula (6). In Formula (6), N represents the number of classes of objects in the dataset, which is set to 3 in this paper.

(5) $$AP=\int_{0}^{1}P\left(R\right)dR$$

(6) $$mAP=\frac{\sum_{i=1}^{N}AP_{i}}{N}$$

The loss value reflects the convergence status of the model during the training process. The loss function used in this paper is represented by Formula (7). The lower the loss function of the model during training, the better the robustness of the model. At the same time, observing the loss curve of the model during training can help to determine whether the model exhibits divergence or overfitting phenomena. In Formula (7), $L_{cls}$ represents the classification loss, $L_{reg}$ represents the localization loss, $L_{obj}$ represents the confidence loss, λ represents the balance coefficient of the localization loss, which is set to 5 in this paper, and $N_{pos}$ represents the number of anchor points classified as positive samples. Formula (8) presents the concrete implementation of the classification loss, where *N* stands for the number of samples, *K* represents the number of categories, and $P_{ic}$ indicates the probability of the ith sample belonging to category c. Equation (9) delineates the precise implementation of the confidence loss, where *N* denotes the number of samples, $y_{i}$ signifies the category to which the ith sample belongs, and $p_{i}$ stands for the predicted value of the ith sample. Equation (10) portrays the precise realization of the regression loss, where GT signifies the actual border, and Pred represents the prediction border.

(7) $$Loss=\frac{L_{cls}+\lambda L_{reg}+L_{obj}}{N_{pos}}$$

(8) $$L_{cls}=-\frac{1}{N}\sum_{i=1}^{N}\sum_{i=1}^{K}y_{ic}log\left(p_{ic}\right)$$

(9) $$L_{obj}=-\frac{1}{N}\sum_{i=1}^{N}\left[y_{i}log\left(p_{i}\right)+\left(1-y_{i}\right)log\left(1-p_{i}\right)\right]$$

(10) $$L_{reg}=-In\frac{Intersection(GT,Pred)}{Union(GT,Pred)}$$

### 4.3. Analysis of Experimental Results

#### 4.3.1. Comparison of YOLOX and ShuffYOLOX

After training the proposed model on the KITTI dataset, to evaluate its performance more accurately, we used the experimental results of YOLOX as the baseline and compared the experimental results of ShuffYOLOX and YOLOX through Table 2.

Based on the experimental results in Table 2, it can be observed that the detection accuracy of ShuffYOLOX is close to that of YOLOX in the Car, Truck, Van, and Pedestrian categories, and increases by almost 0.01% in the Tram category compared to YOLOX. Overall, ShuffYOLOX’s mAP@50 and mAP@5095 decreased by 0.1% and 1%, respectively, compared to YOLOX, but its FPS increased by 65%. In summary, ShuffYOLOX greatly improves the detection speed while maintaining a similar level of detection accuracy to YOLOX.

**Figure 9:**
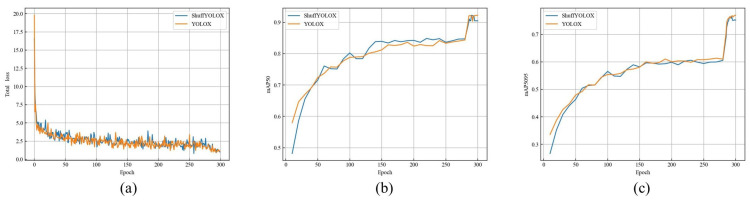
(**a**) is loss curve plot, (**b**) is mAP50 curve plot, (**c**) is mAP5095 curve plot.

To further compare the superiority of our proposed method, we compared the training loss curve of ShuffYOLOX (shown in Figure 9a) and the mAP change curve (shown in Figure 9b,c) with the original YOLOX model.

Based on the results shown in Figure 9a, the changes in the loss values of ShuffYOLOX and YOLOX for each epoch can be clearly observed, and the convergence of the loss values indicates that the model did not experience divergence or overfitting during the entire training process, demonstrating the effectiveness of the ShuffYOLOX model structure. In addition, a faster convergence of the loss curve indicates a higher learning efficiency of the algorithm, and it can be seen from Figure 9a that the convergence speed of the loss curve of ShuffYOLOX is similar to that of YOLOX, indicating that ShuffYOLOX has a learning efficiency similar to YOLOX, but with significantly reduced model parameters, resulting in a much higher detection speed than YOLOX. After 300 epochs, the loss stabilized at around 1.2, indicating that the model training has reached the optimal state. In Figure 9b,c, the evaluation accuracy indicators are mAP@50 and mAP@5095, respectively. Upon analyzing the mAP@50 curve in Figure 9b, it is evident that ShuffYOLOX outperforms YOLOX in terms of mAP50 in the range of 50–250 epochs, suggesting that ShuffYOLOX exhibits superior early-stage learning efficiency as compared to YOLOX. Moreover, an observation of the mAP@5095 curve in Figure 9c reveals that the precision change curves for ShuffYOLOX and YOLOX are approximately the same. This indicates that ShuffYOLOX can achieve detection accuracy requirements that are roughly comparable to those of YOLOX, thus laying a foundation for high-precision requirements in autonomous driving tasks.

**Figure 10:**
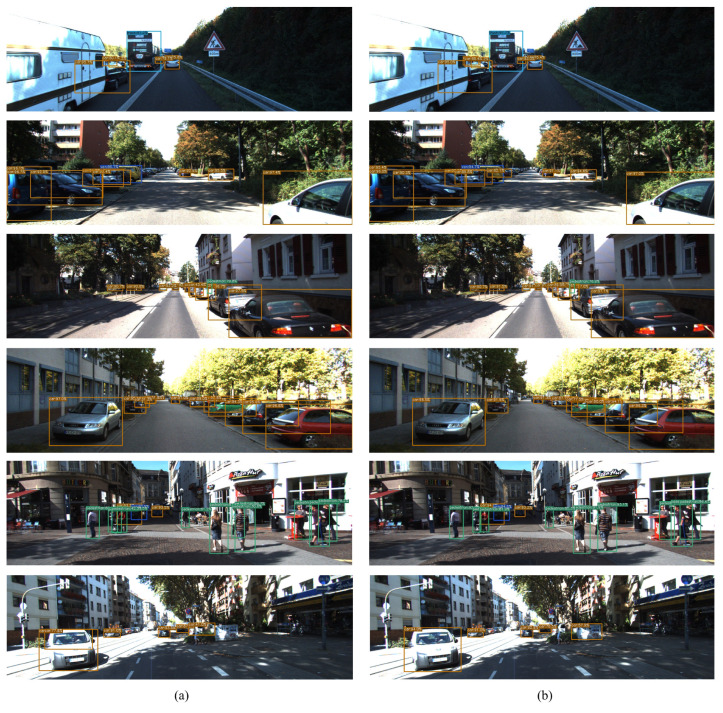
Visualization of detection results, (**a**) is YOLOX, (**b**) is ShuffYOLOX.

According to the visualization results shown in Figure 10, each detection box represents the actual predicted object position by the model, and the top of the detection box indicates the predicted class and confidence. Meanwhile, different colors of detection boxes also represent different objects, with orange, royal blue, sky blue, and green boxes indicating the detection of Car, Van, Truck, and Pedestrian, respectively. From the visualization results, the proposed ShuffYOLOX has excellent detection performance and performs well in recognizing small objects. The prediction accuracy is comparable to that of YOLOX, but the model’s efficiency has significantly increased, making it more suitable for autonomous driving scenarios than YOLOX.

#### 4.3.2. Ablation Experiment

In order to better analyze the effects of the ShuffDet and ECA attention modules introduced in this paper on the performance of the YOLOX model, we conducted ablation experiments based on the KITTI dataset. The specific results are shown in Table 3, where “✔” indicates the corresponding improvement strategy.

In Experiment (1), replacing CSPDrk53 with ShuffDet significantly reduced the number of model parameters and increased the detection speed, but also relatively decreased the model detection accuracy. In Experiment (2), we conducted an experimental comparison on the introduction of the ECA attention module, and the results showed that the ECA module only adds a small number of parameters but can increase mAP by 1%. In Experiments (3) and (4), after adding the SPPS module, the model’s parameter volume decreased and FPS increased, indicating that the SPPS module is more lightweight than the original SPP module. In Experiments (0) and (5), it can be observed that the model’s accuracy was improved after adding the ECA and SPPS modules, indicating that the improvements made in this paper for YOLOX are meaningful and achieved a balance between detection speed and accuracy. Moreover, they have better applicability in the autonomous driving scenario and are more conducive to deployment on autonomous driving terminal devices.

The results show that although ShuffYOLOX reduces 0.07% mAP compared to YOLOX, it significantly reduces the model’s parameter count, reducing the model size by 34.57%, decreasing Gflops by 42.19%, and increasing FPS by 65%. This is attributed to the strict adherence to lightweight design principles in the ShuffYOLOX model architecture, which features a more lightweight design and smaller memory access count (MAC), leading to a significant increase in detection speed.

#### 4.3.3. Result Comparison with other Detection Algorithms

To better validate the effectiveness and scientificity of the proposed algorithm in this paper, we compared the ShuffYOLOX algorithm with current mainstream one-stage object detection algorithms. The evaluation criteria for the model performance were chosen as average precision (mAP), model parameters, and frames per second (FPS). The comparison results are shown in Table 4.

Based on the outcomes in Table 4, we note the following. Firstly, we observe that the SSD300 model, using VGG-16 as its backbone, possesses notably fewer model parameters compared to other single-stage detection models. However, its accuracy falls short of meeting the high precision requirements for autonomous driving. Secondly, we discover that RetinaNet achieves an mAP of 88.68%, with a modest 37.23 M model parameter count. Yet, its detection speed lags significantly, failing to meet the promptness demands of autonomous driving tasks. Within the YOLO series, YOLOV8 stands out with a peak detection accuracy of 93.38%, yet its reliance on the c2f architecture leads to slower network inference speeds. Although YOLOP exhibits remarkable results in multitasking scenarios, its performance in detection tasks is moderate. Lastly, while YOLOV5 and YOLOV8 excel in mAP benchmarks, our proposed ShuffYOLOX strikes the optimal equilibrium between efficiency, model parameters, and accuracy, excelling comprehensively across all dimensions of autonomous driving tasks. In summary, ShuffYOLOX outperforms alternative algorithms across the entirety of KITTI dataset-based performance metrics.

### 4.4. Experiment on Edge Computing Devices Detection

**Figure 11:**
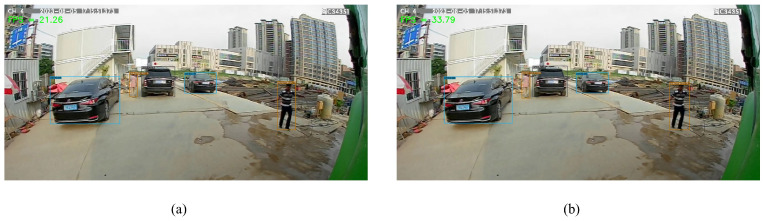
Visualization of inference results on edge computing devices, (**a**) is YOLOX-Nano, (**b**) is ShuffYOLOX-Nano.

In order to validate the efficacy of our proposed algorithm on edge computing devices, we conducted comparative experiments on the Nvidia Jetson Nano 4GB version. Given the limited computational power of edge computing devices, we selected YOLOX-Nano as the control group. Notably, YOLOX-Nano exhibited an inference speed of 21 FPS, whereas ShuffYOLOX-Nano demonstrated an inference speed of 33 FPS. The former’s power consumption stood at 10W, while the latter hovered around 7 W. The experimental outcomes are depicted in Figure 11. The test video was captured via an onboard camera, with a video size of 800 × 480. Clearly discernible is the fact that our proposed ShuffYOLOX can effectively conduct real-time operations on edge computing devices while maintaining commendable detection precision. In comparison to YOLOX, there is a 12 FPS boost, achieving the real-time detection criterion of 30 frames per second, further substantiating the algorithm’s prowess within the realm of autonomous driving scenarios.

## 5. Conclusions

Based on YOLOX, this paper proposes a one-stage object detection algorithm for autonomous driving. In order to improve the efficiency of the YOLOX algorithm in autonomous driving tasks, we have improved the YOLOX algorithm based on lightweight principles and attention mechanisms, such as the lightweight feature extraction network ShuffDet and the attention module ECA. We conducted ablation experiments and result analysis on several different object detection algorithms on the KITTI dataset. The experimental results show that the proposed ShuffYOLOX achieves an accuracy of 92.20% on the KITTI dataset, reducing the model complexity by 40% compared to the original YOLOX, and increasing the model detection speed by 65%. Compared with other object detection algorithms, ShuffYOLOX achieves better detection performance in terms of model lightweightness and accuracy. Its lightweight feature enables efficient deployment on resource-constrained in-vehicle systems, while its high efficiency provides security for complex driving environments in autonomous driving. Our research provides some reference value for future lightweight research on object detection algorithms.

We applied our method to the latest YOLO series network, YOLOV8, achieving noteworthy results. The original YOLOV8’s c2f architecture involves a substantial number of split operations that impede parallelism, leading to slower inference speeds. After substituting the base network with ShuffDet, the inference speed experienced significant acceleration, a 63% enhancement over the original YOLOV8. Simultaneously, YOLOV8’s implementation of the impressive TaskAlignedAssigner positive sample allocation strategy, along with the incorporation of Distribution Focal Loss, markedly elevated its detection accuracy. Upon integrating our proposed approach, detection accuracy closely resembles that of the original YOLOV8, achieving a 93.21% accuracy on the KITTI dataset. This further underscores the universal applicability of our method, serving as a versatile framework for lightweight network design. Our next research goal is to combine the method we have introduced with model compression techniques such as pruning and knowledge distillation, and apply them to object detection tasks. Through the introduction of a multi-stage model compression approach, we hope to further enhance model compression efficiency. Concurrently, we need to comprehensively consider relevant hardware conditions. For example, a key focus of our next research phase is to determine whether our proposed algorithm can effectively run on edge computing devices, especially in extreme weather conditions such as high temperatures.

## Figures and Tables

**Table 1:** Experimental equipment and environmental settings.

Parameter	Configuration
CPU	Intel Core i7-12700KF
GPU	NVIDIA GeForce RTX 3070Ti (8 GB)
CUDA version	CUDA 11.7
Python version	Python 3.9.13
Deep learning framework	Pytorch 2.0.0
Operating system	Ubuntu 22.04.2 LTS

**Table 2:** The experimental results of YOLOX and ShuffYOLOX.

Algorithm	Car	Truck	Tram	Van	Pedestrian	mAP@50	mAP@5095	FPS
YOLOX-L	0.907	0.999	0.989	0.905	0.812	0.923	0.770	68.9
ShuffYOLOX	**0.908**	0.995	**0.997**	0.902	0.808	0.922	0.760	**113.9**

**Table 3:** Results of ablation experiments.

Method	ShuffDet	ECA	SPPS	mAP/%	Params/M	GFLOPs	FPS
YOLOX-L(0)				92.27	54.15	155.69	68.94
YOLOX-L(1)	✔			91.18	34.21	**86.36**	117.81
YOLOX-L(2)	✔	✔		92.16	36.19	91.54	101.67
YOLOX-L(3)	✔		✔	91.35	**33.97**	84.75	**119.18**
YOLOX-L(4)			✔	92.29	53.89	153.68	70.13
YOLOX-L(5)		✔	✔	**92.83**	55.31	157.24	66.86
ShuffYOLOX(6)	✔	✔	✔	**92.20**	**35.43**	**89.99**	**113.90**

**Table 4:** Comparison with other algorithms.

Method	Backbone	Input Size	mAP/%	Params/M	FPS
YOLOv3 [11]	Darknet-53	608 × 608	87.37	61.53	42.14
YOLOv4 [39]	CSPDarknet-53	608 × 608	90.72	64.36	47.58
YOLOv5-L	CSPDarknet-53	640 × 640	92.63	46.52	70.21
YOLOv8-L	CSPDarknet-53-c2f	640 × 640	**93.38**	43.74	65.19
YOLOP [40]	CSPDarknet-53	640 × 640	92.16	51.36	43.58
SSD300 [9]	VGG-16	300 × 300	83.47	**24.26**	47.72
RetinaNet [41]	ResNet-50	500 × 500	88.68	37.23	36.53
YOLOX-L [18]	CSPDarknet-53	640 × 640	**92.27**	54.15	68.94
ShuffYOLOX	ShuffDet	640 × 640	**92.20**	**35.43**	**113.90**

## Data Availability

The data provided in this study can be publicly accessed on KITTI, with the DOI 10.1109/CVPR.2012.6248074 and reference numbers [1].
